# Design of modified fractional-order PID controller for lower limb rehabilitation exoskeleton robot based on an improved elk herd hybridized with grey wolf and multi-verse optimization algorithms

**DOI:** 10.3389/frobt.2025.1667688

**Published:** 2025-11-27

**Authors:** Noor Sabah Mohammed Ali, Muna Hadi Saleh, Nizar Hadi Abbas

**Affiliations:** 1 Department of Electrical Engineering, College of Engineering, University of Baghdad, Baghdad, Iraq; 2 Department of Electrical Engineering, College of Engineering, University of Wasit, Wasit, Iraq

**Keywords:** modified controller, FOPID controller, MFOPID controller, rehabilitation robots, lower limb, improved algorithm, hybrid algorithm, EHO algorithm

## Abstract

Rehabilitation robots are widely recognized as vital for restoring motor function in patients with lower-limb impairments. A Modified Fractional-Order Proportional-Integral-Derivative (MFOPID) controller is proposed to improve trajectory tracking of a 2-DoF Lower Limb Rehabilitation Exoskeleton Robot (LLRER). The classical FOPID is augmented with a modified control formulation by which steady-state error is reduced and the transient response is sharpened. Controller gains and fractional orders were tuned offline using a hybrid metaheuristic Improved Elk Herd Optimization hybridized with Grey Wolf and Multi-Verse Optimization algorithms (IElk-GM) so that exploration and exploitation are balanced. Superiority over the classical FOPID was demonstrated in simulations under linear and nonlinear trajectories, with disturbances and parametric uncertainty: 0% overshoot was achieved at both hip and knee joints; settling time was reduced from 6.998 s to 0.430 s (hip) and from 7.150 s to 0.829 s (knee); ITAE was reduced from 23.39 to 2.694 (hip) and from 16.95 to 3.522 (knee); and the hip steady-state error decreased from 0.018 Rad to 0.0015 Rad, while the knee steady-state error remained within 0.011 Rad. Control torques remained bounded under linear tracking (<345 N·m at the hip; <95 N·m at the knee) and under nonlinear cosine tracking (<350 N·m at the hip; <100 N·m at the knee). These results indicate that safer, smoother, and more effective robot-assisted rehabilitation can be supported by the proposed controller.

## 1 Introduction

Strokes are among the primary causes of long-term disability and mortality among cardiovascular diseases, often resulting in hemiplegia and severe motor dysfunction (Roth et al., 2020; Wang et al., 2020). Rehabilitation plays a crucial role in restoring motor function in stroke patients, particularly in improving lower limb mobility. Traditional rehabilitation methods typically rely on manual interventions by therapists, which are labour-intensive, subjective, and limited in precision and repeatability (Volpe et al., 2001; Sabah et al., 2021). These limitations have accelerated the development of robotic rehabilitation systems, which provide consistent, repeatable training sessions, real-time monitoring, and objective assessments of motor recovery (Kiyono et al., 2024; Su et al., 2023). Lower limb rehabilitation exoskeletons offer significant promise in supporting patient recovery by facilitating gait training through programmable motion patterns (Aguirre-Ollinger et al., 2024; Zhang, 2025). However, due to the interaction between human limbs and robotic actuators, the system dynamics are extremely non-linear and subject to parametric uncertainties and external disturbances (Torabi et al., 2017). The FOPID controllers are gaining increasingly wider acceptance among control strategies due to their application of fractional integral and fractional derivative terms to produce better performances for nonlinear systems (Abdulwahhab and Abbas, 2020). The inclusion of the fractional integral and derivative orders ( λ and μ ) in the FOPID controller introduces two additional tuning parameters beyond those in the classical PID controller. These additional control orders provide greater flexibility in shaping the control response, enabling more precise adjustment of the system’s dynamic behaviour. However, determining the optimal values for all five parameters, proportional gain ( $K_{P}$ ), integral gain ( $K_{I}$ ), derivative gain ( $K_{D}$ ), integral order ( λ ), and derivative order ( μ ), is a complex task. Improper tuning may lead to degraded performance or even instability, making the controller design process significantly more challenging than that of traditional PID controllers (Vanchinathan and Selvaganesan, 2021).

Single-heuristic metaheuristics (e.g., PSO, GWO, MVO, and standard EHO) frequently suffer from premature convergence and an exploration-exploitation imbalance population diversity collapses early, the search stalls near local minima, and performance becomes hyper-parameter sensitive and landscape dependent. GWO tends to emphasize leader-driven exploitation at the expense of global exploration; MVO provides stochastic global jumps but may converge slowly; and EHO preserves diversity yet can lack late-stage intensification. These drawbacks are critical when tuning the parameters of FOPID/MFOPID. To address this, the proposed IElk-GM hybrid combines Improved EHO (diversity preservation + elitism) with GWO (structured local refinement) and MVO (probabilistic space warping), with adaptive coefficients and elitism to sustain exploration early and accelerate exploitation late reducing stagnation and improving reproducible convergence for MFOPID tuning.

Several researchers have explored FOPID controllers in rehabilitation robotics. For instance:

(Ayas et al., 2016) Proposed a FOPID controller for enhanced trajectory tracking of a 2-DoF parallel ankle rehabilitation robot in the presence of disturbances. Their results demonstrate that the optimally tuned FOPID controller considerably enhances tracking performance of the ankle rehabilitation robot in the presence of external disturbances and reduces more steady-state tracking errors than the optimally tuned PID controller.

(Wang et al., 2022) Proposed a fractional order $PI^{\lambda }D^{\mu }$ for tracking and control of an innovative rehabilitation robot using the Improved Ions Motion Optimization Back Propagation (IIMO-BP) neural network method. Their results demonstrate that the robust control strategy guarantees a stable environment for rehabilitation training, as well as the rationality and effectiveness of trajectory planning.

(Faraj et al., 2023) proposed an Adaptive Optimal Fractional-order Super-Twisting Sliding-Mode (AOFSTSM) controller for lower-limb rehabilitation under constrained motion with ground contact, combining fractional operators with a super-twisting algorithm for chatter mitigation and an adaptive bound estimator; controller gains were tuned via Grey Wolf Optimization (GWO) algorithm. Their results show robust tracking under disturbances and parametric uncertainties. In contrast, the present work adopts a different control paradigm: a Modified Fractional-Order PID (MFOPID) with nonlinear error shaping that yields continuous control torques (no discontinuous switching), aiming at smoothness and patient comfort together with embedded simplicity. The MFOPID gains and fractional orders are tuned offline using hybrid IElk-GM optimizer (Improved Elk Herd Optimization + Grey Wolf Optimization + Multi-Verse Optimization), which improves exploration-exploitation balance relative to single-population GWO while keeping the run-time controller fixed-structure (no online adaptive laws). This positions our contribution as complementary to AOFSTSM: while AOFSTSM prioritizes invariance through sliding-mode mechanisms, our MFOPID targets overshoot-free, smooth transients and low implementation burden. To operationalize this contrast, Section 6 reports standard time-domain indices Table 6, a qualitative smoothness summary Table 7 and an implementation-complexity comparison Table 8. (Where numeric data are unavailable in (Faraj et al., 2023), comparisons are made from the published plots).

(Ning et al., 2024) Proposed a multi-objective inverse kinematics model for redundant rehabilitation robots, solved using an Improved Equilibrium Optimization (IEO) algorithm. Their results show higher accuracy, robustness, and more human-like rehabilitation trajectories compared to conventional optimization methods.

(He et al., 2024) Proposed a Fractional-Order ultra-local model-based Finite-Time Robust Controller (FO-FTRC) for trajectory tracking of rehabilitation robots under uncertainties and disturbances. Their results demonstrate that the model-free robust strategy ensures accurate tracking performance and superior robustness compared to conventional adaptive and sliding mode methods.

(Xie et al., 2025) Proposed a motion control framework for lower limb rehabilitation robots by integrating optimal S-type trajectory planning, zero-force control using the LuGre friction model, and a singular perturbation-based control strategy. Their results demonstrate that the proposed approach significantly improves trajectory smoothness, tracking accuracy, and robustness against external disturbances, thereby providing patients with safer and more effective rehabilitation training.

Despite the diversity of fractional-order control strategies applied in rehabilitation robotics, certain limitations remain unresolved particularly regarding adaptability to nonlinear trajectory tracking, dynamic patient-robot interaction, and robustness against model uncertainties. Most prior studies have concentrated on parameter optimization of FOPID controllers while retaining a fixed control structure, which inherently restricts their flexibility in complex rehabilitation scenarios.

To address these challenges, this paper introduces a Modified Fractional-Order PID (MFOPID) controller that extends the classical FOPID by incorporating a nonlinear error formulation. This structural enhancement is designed to improve transient response, suppress overshoot, and minimize steady-state error, thereby offering a more effective control solution for lower-limb rehabilitation robots. The MFOPID design is inspired by the conventional FOPID formulation in (Vanchinathan and Selvaganesan, 2021), but it incorporates structural modifications that enhance control performance in the context of rehabilitation robotics. To further improve the controller’s effectiveness, an improved hybrid metaheuristic algorithm, the Improved Elk Herd Optimization hybridized with Grey Wolf Optimization and Multi-Verse Optimization (IElk-GM), is employed for parameter tuning. By combining the exploration-exploitation capabilities of three nature-inspired optimizers, the IElk-GM algorithm achieves faster convergence and improved robustness compared to individual optimization methods. The proposed MFOPID controller is implemented on a 2-DoF lower limb rehabilitation robot modelled using dynamic equations that capture the biomechanical behaviour of a human lower limb during walking. Lyapunov stability is used for stability analysis of both system joints under the dynamic equations of the robot’s control closed-loop.

The key contributions can be described as follows: a modify FOPID controller structure has been suggested to improve steady and transient characteristics in the lower limb rehabilitation tasks, an improved hybrid metaheuristic algorithm (IElk-GM) is developed for efficient and accurate controller parameter tuning, the proposed controller is validated through dynamic simulations under both linear and non-linear trajectory conditions, with disturbances and uncertainties, a comparative performance analysis is conducted against the classical FOPID controller to demonstrate the improvements in tracking accuracy, stability, and control smoothness.

This paper is organized into seven sections. Section 2 presents the mathematical significance of the proposed framework. Section 3 describes the dynamic mathematical model of the two-link LLRER. Section 4 details the design of the modified fractional-order PID controller. Section 5 introduces the hybrid optimization algorithm used for controller tuning. Section 6 discusses the simulation results under various conditions. Finally, the last section concludes and proposes future work areas.

## 2 Mathematical significance of the proposed framework

The proposed framework presents substantial mathematical contributions to the field of intelligent control and optimization. Firstly, the Modified Fractional Order PID (MFOPID) controller introduces a non-linear error formulation that extends the classical FOPID structure by incorporating additional control parameters and non-linear terms. This modification enables finer control of system dynamics, which is analytically validated through Lyapunov-based stability analysis. The stability proof ensures that the proposed controller achieves global convergence with reduced overshoot and improved transient performance. Secondly, the hybrid IElk-GM algorithm constitutes a mathematically rich integration of three nature-inspired metaheuristics: Improved Elk Herd Optimization (EHO), Grey Wolf Optimization (GWO), and Multi-Verse Optimization (MVO). Each component contributes distinct mathematical operators, leadership-based exploration, social hierarchy modelling, and probabilistic space warping, resulting in a balanced global-local search mechanism. The formulation of the algorithm includes adaptive control coefficients, elitism preservation, and probabilistic wormhole operations, all of which are mathematically defined and governed by time-varying parameters. Moreover, the control design and optimization process are formalized through the minimization of a Time Integrated Absolute Error $\left(ITAE\right)$ cost function, which is a classical yet mathematically rigorous performance criterion. The convergence behaviour of the algorithm and the smoothness of the control response further highlight the analytical soundness of the method. Taken together, the controller design and the optimization algorithm proposed in this study form a cohesive mathematical model for intelligent control systems, providing both theoretical insights and practical performance enhancements in non-linear robotic rehabilitation systems.

## 3 Dynamic model of LLRER

### 3.1 Physical exoskeleton and control architecture

The target platform is configured as a planar 2-DoF lower limb rehabilitation exoskeleton operating in the sagittal plane. Hip flexion extension is denoted by $\theta_{1}$ and knee flexion extension by $\theta_{2}$ ; the joint coordinate vector is $\theta ={\left[\theta_{1}\;\theta_{2}\right]}^{T}$ with velocities $\dot{\theta }$ and accelerations $\ddot{\theta }$ . Rigid thigh and shank frames are attached to the patient via adjustable cuffs and quick-release straps, and link lengths are adjusted to the user’s anthropometry. Typical range of motion envelopes are considered to guide control and safety limits ( hip≈−20° to+120°,knee≈0° to+130° ), and mechanical end-stops are provided near the extremes to prevent over-travel. Each joint is actuated by an electric drive with high-ratio transmission (e.g., harmonic or planetary gearing), by which motor torque is amplified while reflected inertia is kept within clinically acceptable limits. Optional series elasticity or software torque limiting is employed to improve comfort during therapy. Joint-level torque and speed limits are enforced in firmware to maintain operation within safe bounds consistent with the limits used in the results section. Joint angles are measured using high-resolution absolute encoders, and joint velocities are obtained by numerical differentiation with appropriate filtering. Drive currents are monitored for torque estimation; inertial measurement units may be mounted on the thigh and shank for segment level orientation, and foot-contact sensing (e.g., insole force sensors) can be used for gait-phase or state detection. All safety critical signals (emergency stop, over-current, over-temperature) are handled by hardware interlocks in parallel with software supervision. Control is executed on a real-time embedded controller. A cascaded structure is adopted: an inner current/torque loop runs at high frequency to regulate actuator torque; a joint-position loop implements the MFOPID at a lower, yet real-time, rate; and a high-level trajectory generator with a safety supervisor coordinates task execution and enforces limits. The MFOPID parameters (gains and fractional orders) are tuned offline using a hybrid metaheuristic Improved Elk Herd Optimization hybridized with Grey Wolf and Multi-Verse Optimization algorithms (IElk-GM) so that exploration and exploitation are balanced during tuning while runtime complexity remains minimal.

### 3.2 Dynamic modeling

**FIGURE 1:**
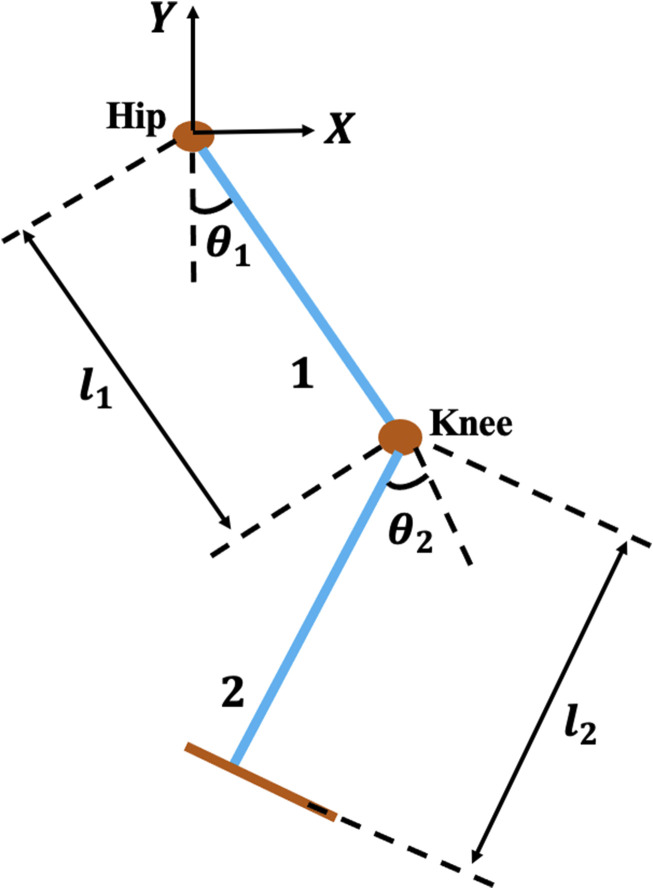
2-DoF hip-knee schematic for the LLRER.

The LLRER considered in this study is a planar 2-DoF structure consisting of two rigid links and two revolute joints, which correspond to the hip and knee joints of the human body. This configuration is designed to facilitate flexion and extension movement in the sagittal plane, thereby enabling gait rehabilitation for stroke and mobility-impaired patients (Al Rezage and Tokhi, 2016). The mechanical structure of the robot is shown in Figure 1. A dynamic model based on the anthropometric features of a human lower limb is used to describe the mobility of the robot. The model assumes a subject with a body mass of 74 kg and height of 1.69 m, with segment properties obtained from winter’s anthropometric data (Alshatti, 2019; Winter 2009). The robot dynamics are derived using the Euler-Lagrange method, capturing the effects of joint inertia, Coriolis and centrifugal forces, gravitational torque, control inputs, and external disturbances.

The general 2-DoF dynamics are given in Equation 1:

$$M\left(\theta \right)\ddot{\theta }+C\left(\theta ,\dot{\theta }\right)\dot{\theta }+G\left(\theta \right)+d\left(t\right)=u\left(t\right)$$ (1)

The angle, angular velocity, and acceleration of a robot joint vector are denoted by the variables $\theta ,\dot{\theta }$ , and $\ddot{\theta }$ , respectively. For every inertia $M\left(\theta \right)$ , Coriolis, and centrifugal torque *C* (*θ*, $\dot{\theta }$ ) ∈ $R^{\left(2\times 2\right)}$ in human limb matrices. The one-dimensional vector of the torque of gravity $\left(G\left(\theta \right)\right)$ is ∈ $R^{\left(2\times 1\right)}$ , the control signal is represented by $u\left(t\right)$ , and the vector of external disturbances is $d\left(t\right)\in R^{n}$ .

The dynamics of the robot are indicated by Equation 2:

$$\left[\begin{array}{cc} M_{11} & M_{12}\\ M_{21} & M_{22} \end{array}\right]\left[\begin{array}{c} {\ddot{\theta }}_{1}\\ {\ddot{\theta }}_{2} \end{array}\right]+\left[\begin{array}{cc} C_{11} & C_{12}\\ C_{21} & C_{22} \end{array}\right]\left[\begin{array}{c} {\dot{\theta }}_{1}\\ {\dot{\theta }}_{2} \end{array}\right]+\left[\begin{array}{c} G_{1}\\ G_{2} \end{array}\right]=\left[\begin{array}{c} {u\left(t\right)}_{1}\\ {u\left(t\right)}_{2} \end{array}\right]$$ (2)

The components of the inertia matrix M(θ) are depicted in Equation 3:

$$\begin{array}{l} M_{11}=I_{1}+I_{2}+m_{1}{\left(L_{C1}\right)}^{2}+m_{2}{\left(L_{1}\right)}^{2}+m_{2}{\left(L_{C2}\right)}^{2}+2m_{2}L_{1}L_{C2}\;\cos\left(\theta_{2}\right)\\ M_{12}=M_{21}=I_{2}+m_{2}{\left(L_{C2}\right)}^{2}+m_{2}L_{1}L_{C2\;}\cos\left(\theta_{2}\right)\\ M_{22}=I_{2}+m_{2}{\left(L_{C2}\right)}^{2} \end{array}$$ (3)

The elements of $C\left(\theta ,\dot{\theta }\right)$ are determined by Equation 4:

$$\begin{array}{l} C_{11}=-m_{2}L_{1}L_{C2}\;\sin\left(\theta_{2}\right){\dot{\theta }}_{2}\\ C_{12}=-m_{2}L_{1}L_{C2}\;\sin\left(\theta_{2}\right)\left({\dot{\theta }}_{1}+{\dot{\theta }}_{2}\right)\\ C_{21}=m_{2}L_{1}L_{C2}\;\sin\left(\theta_{2}\right){\dot{\theta }}_{1}\\ C_{22}=0 \end{array}$$ (4)

The parameters of the gravitational vector $G\left(\theta \right)$ are specified by Equation 5:

$$\begin{array}{l} G_{1}=m_{1}L_{C1}g\;\sin\left(\theta_{1}\right)+m_{2}gL_{1}\;\sin\left(\theta_{1}\right)+m_{2}gL_{C2}\sin\left(\theta_{1}+\theta_{2}\right)\\ G_{2}=m_{2}gL_{C2}\cos\left(\theta_{1}+\theta_{2}\right) \end{array}$$ (5)

The variables of these equations are delineated by specific parameters presented in Table 1.

**TABLE 1:** Physical parameters and variables of LLRER.

Parameters	Value
Length of link 1 (L_1_)	0.54 m
Length of link 2 (L_2_)	0.48 m
Link (1) centre of mass ( $L_{C1}$ )	0.2338 m
Link (2) centre of mass ( $L_{C2}$ )	0.241 m
Link 1 mass (m_1_)	8 Kg
Link 2 mass (m_2_)	3.72 Kg
Link 1 inertia (I_1_)	0.42 kg.m^2^
Link 2 inertia (I_2_)	0.07 kg.m^2^
Acceleration by gravity (g)	9.8 m/s^2^
Link 1 angular displacement ( $\theta_{1}$ )	N/A Rad
Link 2 angular displacement ( $\theta_{2}$ )	N/A Rad
Link 1 angular velocity ( ${\dot{\theta }}_{1}$ )	N/A Rad/s
Link 2 angular velocity ( ${\dot{\theta }}_{2}$ )	N/A Rad/s
Angular acceleration ( $\ddot{\theta }$ )	N/A Rad/s^2^

## 4 Fractional order PID controller (FOPID) design

**FIGURE 2:**
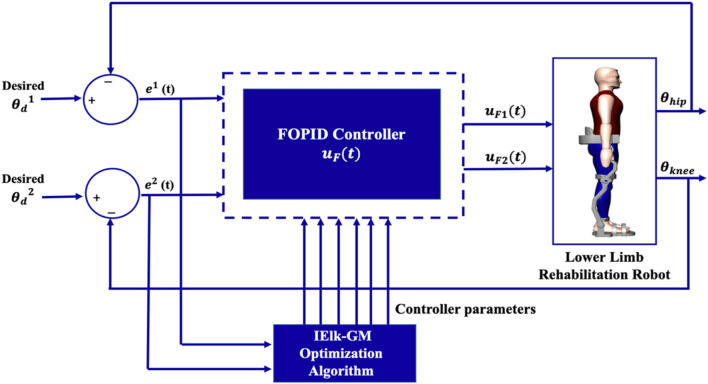
The block diagram of the FOPID.

The structures of the Adaptive PID and FOPID controllers suggested in (Vanchinathan and Selvaganesan, 2021; Noordin et al., 2023) are used for building a controller for the two-link LLRER. Figure 2 illustrates a block diagram of the designed controller.

Equation 6 defines the Alpha function as follows:

$$\gamma_{L}\left(t\right)=H_{PL}e_{L}\left(t\right)+H_{DL}{\dot{e}}_{L}\left(t\right)$$ (6)

where L = 1, 2 is the link number. $e_{L}$ is the instantaneous error, which shows the difference between the current desired trajectory $\theta_{dL}$ and actual output $\theta_{L}$ of link ( L ) as in Equation 7:

$$e_{L}=\theta_{dL}-\theta_{L}$$ (7)

Equation 8 describes the control law for this controller:

$$u_{FL}\left(t\right)=M\left(\theta ,\dot{\theta }\right)*u_{FOPIDL}\left(t\right)$$ (8)

Also, $u_{FOPIDL}\left(t\right)$ is defined in Equation 9:

$$u_{FOPIDL}\left(t\right)=K_{PL}{\left(t\right)e}_{L}\left(t\right)+K_{IL}\left(t\right){\left(\int_{0}^{t}\right)}^{\lambda L}e_{L}\left(t\right)dt+K_{DL}\left(t\right)\frac{d^{\mu L}}{{dt}^{\mu L}}e_{L}\left(t\right)$$ (9)

where the parameters $K_{PL}\left(t\right),K_{IL}\left(t\right),and\;K_{DL}\left(t\right)$ are those obtained from Equation 10 through Equation 12:

$$K_{PL}\left(t\right)=\int \dot{K_{PL}}\left(t\right)⟹\dot{K_{PL}}\left(t\right)=\beta_{1L}\;\gamma_{L}\left(t\right)\;e_{L}\left(t\right)$$ (10)

$$K_{IL}\left(t\right)=\int \dot{K_{IL}}\left(t\right)⟹\dot{K_{IL}}\left(t\right)=\beta_{2L}{\;\gamma }_{L}\left(t\right)\int e_{L}\left(t\right)\;dt$$ (11)

$$K_{DL}\left(t\right)=\int {\dot{K}}_{DL}\left(t\right)⟹\dot{K_{DL}}\left(t\right)=\beta_{3L}{\;\gamma }_{L}\left(t\right)\;\dot{e_{L}}\left(t\right)$$ (12)

where $\beta_{1L}$ , $\beta_{2L}$ , and $\beta_{3L}$ stand for positive learning rate. For the controller gains, choosing suitable learning rates and initial values is essential.

The IElk-GM algorithm will determine the optimal parameters of the FOPID controller $u_{FOPID}\left(t\right)$ for link1 ( $(H_{P1},H_{D1},$ $\beta_{11},\beta_{21},\beta_{31},\lambda_{1},and\;\mu_{1})$ , and link2 ( $\left.H_{P2},H_{D2},\beta_{12},\beta_{22},\beta_{32},\lambda_{2},and\;\mu_{2}\right)$ .

### 4.1 Modified fractional order PID controller (MFOPID) design

To improve performance and efficiency, a modified fractional order PID controller is suggested to reduce overshoot and steady-state error. Equation 13 shows the modified alpha function from Equation 6:

$$\gamma_{L}\left(t\right)=H_{PL}e_{L}\left(t\right)+H_{IL}\int e_{L}\left(t\right)dt+H_{DL}{\dot{e}}_{L}\left(t\right)$$ (13)

Accordingly, Equation 8 is to be modified in Equation 14 for the control law:

$$u_{modL}\left(t\right)=M\left(\theta ,\dot{\theta }\right)\left(u_{FOPIDL}\left(t\right)+u_{aux}\left(t\right)\right)$$ (14)

where Equation 15 defines $u_{aux}\left(t\right):$

$$u_{aux}\left(t\right)=\tanh\;\left(\gamma_{L}\left(t\right)\right)*K_{modL}$$ (15)

The IElk-GM algorithm is utilized to get system parameters for the MFOPID controller. The control goals are accomplished by the way this algorithm’s fitness function is set up. The candidate Lyapunov function is given by Equations 16, 17 as follows:

$$V_{L}\left(t\right)=\frac{1}{2}\;{\gamma_{L}}^{2}\left(t\right)$$ (16)

$$\dot{V_{L}}\left(t\right)=\gamma_{L}\left(t\right)\dot{\gamma_{L}},where\;\left(t\right)<0$$ (17)

when $V_{L}<0$ it is guaranteed that $\gamma_{L}\to 0$ as t→∞ . From Equation 13, it can be written:

$$\dot{\gamma_{L}}\left(t\right)=H_{PL}{\dot{e}}_{L}\left(t\right)+H_{IL}\int {\dot{e}}_{L}\left(t\right)dt+H_{DL}\ddot{e_{L}}\left(t\right)$$ (18)

Substituting Equation 18 into Equation 17:

$$\dot{V_{L}}\left(t\right)=\gamma_{L}\left(t\right)\left[H_{PL}{\dot{e}}_{L}\left(t\right)+H_{IL}\int {\dot{e}}_{L}\left(t\right)dt+H_{DL}\ddot{e_{L}}\left(t\right)\right]<0$$ (19)

The negativity condition used in the stability proof is expressed in Equation 19.

Since $V_{L}\left(t\right)$ ≥0 and $\dot{V_{L}}\left(t\right)\leq 0$ , hence, according to the Lyapunov direct method, the system is Lyapunov globally stable. Moreover, due to the structure of $\gamma_{L}\left(t\right)$ , the convergence of $e_{L}\left(t\right)\to 0$ is smooth and without overshoot, as confirmed in simulation.

The IElk-GM algorithm is also used to calculate the optimal parameters of the controller $u_{\mathrm{mod}}\left(t\right)$ of link1 ( $\left.H_{P1},H_{I1}{,H}_{D1},\beta_{11},\beta_{21},\beta_{31},\lambda_{1},\mu_{1},and\;K_{\mathrm{mod}1}\right)$ , and link2 ( $H_{P2},{H_{I2},H}_{D2},\beta_{12},\beta_{22},\beta_{32},\lambda_{2},\mu_{2},and{\;K}_{\mathrm{mod}2}).$

### 4.2 Theoretical gaps in stability analysis

The nominal Lyapunov analysis in Section 4 establishes stability under ideal conditions. In rehabilitation, however, three non-ideal effects are unavoidable: (i) actuator saturation (torque limits and anti-windup), (ii) small I/O delays from sensing/actuation, and (iii) patient-induced disturbances (matched torques at the joints). Let $u_{c}$ denote the MFOPID control before limits and $u=sat\left(u_{c}\right)$ the applied torque after saturation. Let $\Delta u:=u-u_{c}$ be the saturation mismatch; $\tau_{d}\left(t\right)$ bounded patient torque disturbances; and $\tau \in \left[0,\tau_{\max}\right]$ a constant small delay in the loop (sensing/actuation). We use the same Lyapunov candidate $V\left(\cdot \right)$ as in Section 4 and the same error vector e . Closed-loop maps are locally Lipschitz.

#### 4.2.1 Actuator saturation

Assume the saturator is sector-bounded. The static nonlinearity $sat\left(\cdot \right)$ lies in sector $S\left(K\right):$ for some $K\in \left(0,1\right]$ , $0\leq {\left(v-sat\left(v\right)\right)}^{⊤}\;v\leq K{\left\|v\right\|}^{2}\;\forall v.$ Equivalently, $\left\|\Delta u\right\|\leq K\left\|u_{c}\;\right\|$ .

The MFOPID closed loop admits constants $\alpha ,\beta_{u}>0$ such that along solutions

$$\dot{V}\leq -\alpha {\left\|e\right\|}^{2}+\beta_{u}{\left\|\Delta u\right\|}^{2}$$

Hence the system is Input-to-State Stable (ISS) w.r.t. the input Δu ; the tracking error is ultimately bounded with a radius that scales monotonically with K . In practice, torque limiting and anti-windup (as used in our simulations) keep K small, so the residual set is tight and nominal convergence is recovered away from the limits. A standard anti-windup clamp on the fractional integral action preserves the above bound and prevents drift when u sticks to its limits.

#### 4.2.2 Small I/O delays

A constant delay $\tau \in \left[0,\tau_{\max}\right]$ affects either sensing or actuation; the delay-free closed loop is exponentially stable in the nominal sense of Section 4. Suppose there exist $\sigma \in \left(0,1\right)$ and η>0 such that the Razumikhin condition holds:

$$V\left(x\left(t\right)\right)\geq \sigma \;V\left(x\left(t-\tau \right)\right)⟹\dot{V}\left(x\left(t\right)\right)\leq -\eta {\left\|e\left(t\right)\right\|}^{2}$$

Then the MFOPID closed loop is robust to delays for all $\tau \leq \tau_{\max}$ (with $\tau_{\max}$ determined by local Lipschitz bounds). Convergence degrades smoothly as τ increases, but boundedness and asymptotic decay to a small residual set are preserved.

#### 4.2.3 Patient-induced disturbances

For bounded matched torque. The disturbance enters the torque channel and satisfies ${\left\|\tau_{d}\right\|}_{\infty }\leq d_{\max}$ . There exist $c_{1},c_{2}>0$ such that

$$\dot{V}\leq -c_{1}{\left\|e\right\|}^{2}+c_{2}{\left\|\tau_{d}\right\|}^{2}$$

Therefore, the closed loop is ISS w.r.t. $\tau_{d}$ , i.e., $\left\|e\left(t\right)\right\|\leq \beta \left(\left\|e\left(0\right)\right\|,t\right)+\gamma \left(d_{\max}\right)$ , for class-for class- KL and class- K functions β,γ . When the disturbance vanishes, the nominal convergence of Section 4 is recovered.

Under (i-iii) and the nominal hypotheses of Section 4, there exist α>0 and constants $\beta_{u},\beta_{d},\beta_{\tau }>0$ such that

$$\dot{V}\leq -\alpha {\left\|e\right\|}^{2}+\beta_{u}{\left\|∆u\right\|}^{2}+\beta_{d}{\left\|\tau_{d}\right\|}^{2}+\beta_{\tau }\tau^{2}$$

Consequently, the MFOPID closed loop is ISS with respect to saturation mismatch, patient-induced torques, and small delays, and the tracking error is ultimately bounded by a radius that scales with K , $d_{\max}$ , and τ . This upgrades the ideal analysis to practical stability in the sense most relevant to rehabilitation robotics. Keeping the anti-windup gain sufficiently strong (small effective K ), minimizing sensor/actuator latency $\left(\tau \right)$ , and attenuating predictable patient torques (small $d_{\max}$ ) make the residual bound negligible, consistent with the robustness outcomes summarized in Tables 6, 9, and 10 and the time-responses in Figures 10–17. The same bounds apply on the reduced constrained dynamics used for ground-contact walking, making the guarantees directly comparable to Faraj et al. (2023).

Relation to (Faraj et al., 2023), Faraj et al. derive a constrained-motion model for ground contact and prove sliding-mode convergence for their fractional super-twisting controller tuned by GWO, emphasizing invariance against uncertainties and disturbances along the sliding manifold (finite-time/strong robustness on the manifold). While their analysis focuses on constrained dynamics and sliding invariance, it does not explicitly treat actuator saturation or I/O delays. The results above complement that line of work for PID-type continuous control: MFOPID remains non-switching, and its stability is now guaranteed under torque limits, small delays, and bounded patient torques via ISS/ultimate-boundedness. This addresses the practical conditions critical for rehabilitation sessions and aligns the theory with our robustness experiments.

## 5 Optimization algorithm/IElk-GM

Optimization is the selection of the best element, based on some criterion, from a set of available alternatives (Chen et al., 2023). Optimal tuning of the MFOPID controller parameters is critical to ensuring robust trajectory tracking and system stability. To address the challenges of high-dimensional, non-linear optimization inherent in FOPID-based control design, this study proposes an improved hybrid metaheuristic algorithm: Improved Elk Herd Optimization hybridized with Grey Wolf Optimization and Multi-Verse Optimization Algorithms (IElk-GM). Equation 20 provides the Integral Time Absolute Errors (ITAE), the IElk-GM fitness function is:

$$F=ITAE=\int_{0}^{\infty }t\left|e\left(t\right)\right|dt$$ (20)

This cost function penalizes significant errors over time, encouraging fast settling and minimal steady-state deviation.

### 5.1 An improved elk herd optimization algorithm hybridized with grey wolf and multi-verse algorithms (IElk-GM)

The proposed IElk-GM algorithm is a hybrid metaheuristic that integrates three nature-inspired optimization strategies to balance global exploration and local exploitation.

• Improved Elk Herd Optimization (IElk-GM): The algorithm forms the backbone of the search process, offering enhanced population diversity and a structured Herd-based exploration mechanism. Unlike the standard EHO, the improved version incorporates elitism preservation, adaptive parameter control, and balanced harem assignment, which significantly enhance convergence speed and solution quality.
• Grey Wolf Optimization (GWO) is employed to introduce local refinement by simulating leadership-based social hierarchy through α,β,and δ wolves, enabling precise tuning in the vicinity of promising solutions. To avoid stagnation in local optima.
• Multi-Verse Optimization (MVO) is integrated via a stochastic wormhole mechanism, promoting global search through probabilistic space jumps.

**FIGURE 3:**
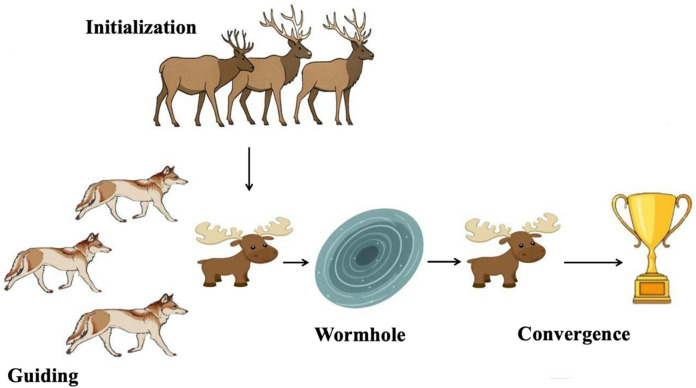
The IElk-GM algorithm phases.

This hybridization of IEHO, GWO, and MVO capitalizes on the strengths of each algorithm: diversity, leadership-based exploitation, and randomness, resulting in a more robust and efficient optimization framework for tuning complex control parameters.

For more information about the Elk Herd Optimization Algorithm, the Grey Wolf Optimization Algorithm, and the Multi-Verse Optimization Algorithm, see (Al-Betar et al., 2024; Mirjalili et al., 2014; Mirjalili et al., 2016).

The overall working process of the proposed IElk-GM algorithm is presented in Figure 3.

#### 5.1.1 Population initialization

Let N be the total population size (elk Herd size), and D be the problem dimensionality (number of decision variables). Each solution vector ${\vec{x}}_{i},\in R^{D}$ is initialized uniformly within the lower ( lb ) and upper ( ub ) bounds of the research space:

$$x_{i}^{d}={lb}_{d}+rand\left(0,1\right)\cdot \left({ub}_{d}-{lb}_{d}\right),\forall_{d}=1,2,\ldots ,D,\text{and }\forall_{i}=1,2,\ldots ,N$$ (21)

The IElk-GM update rules are summarized in Equations 21–35.

Every individual is evaluated by the fitness function $f\left(\underset{x}{\to }\right)$ in order to think about the solutions.

#### 5.1.2 Elitism strategy

To preserve the best performing individuals, the top E individuals are retained across generations, where:

$$E=\left[EliteRate\cdot N\right]$$ (22)

where E is the number of elite individuals preserved per generation.

Let $\left\{{\vec{x}}_{1}\right.,{\vec{x}}_{2},\ldots {\vec{x}}_{E}$ be the elite set such that:

$$f\left({\vec{x}}_{1}\right)\leq f\left({\vec{x}}_{2}\right)\leq \ldots \leq f\left({\vec{x}}_{E}\right)$$ (23)

These elites are directly passed to the next-generation.

#### 5.1.3 Harem assignment (rutting season)

The top B individuals are selected as the number of bulls (leaders):

$$B=\left[B_{r}\cdot N\right]$$ (24)

where $B_{r}$ is the bull rate, $B_{r}\in \left(0,1\right)$ typically in the range [0.1, 0.3].

The remaining ( $\left.N-B\right)$ are designated as harems. The probability of a harem being assigned to a specific bull j is based on inverse fitness:

Pj=1fx→j∑k=1B1fx→k,∀j∈1,…,B (25)

Harems are probabilistically assigned to bulls using a roulette wheel selection mechanism based on $P_{j}$ .

#### 5.1.4 Calving process with GWO

Each bull and its harem generate new offspring (calves). The GWO-inspired model is used to refine calf positions using the top three global solutions, known as Alpha ( $\left({\vec{X}}_{\alpha }\right)$ , Beta ( $\left({\vec{X}}_{\beta }\right)$ , and Delta ( $\left(\vec{X_{\alpha }}\right)$ wolves. The standard GWO equations are:

$${\vec{D}}_{\alpha }=\left|{\vec{C}}_{\alpha }\cdot {\vec{X}}_{\alpha }-\vec{X}\right|$$ (26)

$${\vec{X}}_{1}={\vec{X}}_{\alpha }-{\vec{A}}_{1}\cdot {\vec{D}}_{\alpha }$$ (27)

$${\vec{D}}_{\beta }=\left|{\vec{C}}_{2}\cdot {\vec{X}}_{\beta }-\vec{X}\right|$$ (28)

$${\vec{X}}_{2}={\vec{X}}_{\beta }-{\vec{A}}_{2}\cdot {\vec{D}}_{\beta }$$ (29)

$${\vec{D}}_{\delta }=\left|{\vec{C}}_{3}\cdot {\vec{X}}_{\delta }-\vec{X}\right|$$ (30)

$${\vec{X}}_{3}={\vec{X}}_{\delta }-{\vec{A}}_{3}\cdot {\vec{D}}_{\delta }$$ (31)

where: ${\vec{D}}_{\alpha },{\vec{D}}_{\beta },{\vec{D}}_{\delta }$ is the distance vector between alpha, beta, and delta solution and current, $\vec{X}$ is current solution position, ${\vec{X}}_{1},{\vec{X}}_{2},{\vec{X}}_{3}$ is intermediate updated positions computed based on alpha, beta, delta wolves, ${\vec{C}}_{k}$ is a coefficient vector used in GWO to control the influence of leader wolves (the current best solution) during the position update of a search agent (calf in IElk-GM) defined as ${\vec{C}}_{k}=2\;r_{k}$ where $r_{k}$ is random number sampled from uniform distribution in [0, 1], and ${\vec{A}}_{k}$ is the adaptive control coefficient in GWO defined as ${\vec{A}}_{k}=2a\cdot r_{k}-a$ where a is linearly decreasing parameter from 2 to 0 over iterations defined as $a=2-\frac{2t}{MaxIter}$ where t is current iteration number and MaxIter is maximum number of iterations.

The updated position of the calf is given by:

$${\vec{X}}_{calf}=\frac{1}{3}\left({\vec{X}}_{1}+{\vec{X}}_{2}+{\vec{X}}_{3}\right)$$ (32)

where ${\vec{X}}_{calf}$ is the final updated position of the calf after the GWO-based update.

#### 5.1.5 Wormhole mechanizm via MVO

To introduce global stochasticity and enhance exploration, the MVO wormhole mechanism is applied to each calf with a probability WEP :

$$x_{i,d}^{new}=x_{i,d}\pm TDR\cdot \left({ub}_{d}-{ld}_{d}\right)\cdot N\left(0,1\right)$$ (33)

where: $N\left(0,1\right):$ is standard Gaussian noise. WEP: is wormhole Existence Probability, increasing over time, defined as:

$$WEP={WEP}_{\min}+\left(\frac{t}{MaxIter}\right)\cdot \left({WEP}_{\max}-{WEP}_{\min}\right)$$ (34)

TDR: is travelling Distance Rate, decreasing over time, defined as:

$$TDR=1-\left(\frac{t}{MaxIter}\right)$$ (35)

#### 5.1.6 Population merging and survival selection

The original Herd and the newly generated calves are merged. After sorting all individuals by fitness, the elite solutions E from the previous generation are preserved, and the remaining ( N−E ) individuals are selected from the best-performing candidates in the merged set.

#### 5.1.7 Termination

The algorithm proceeds iteratively until the predefined maximum number of iterations ( $\left.MaxIter\right)$ is reached. At termination, the best solution ${\vec{X}}_{best}$ is returned.

**FIGURE 4:**
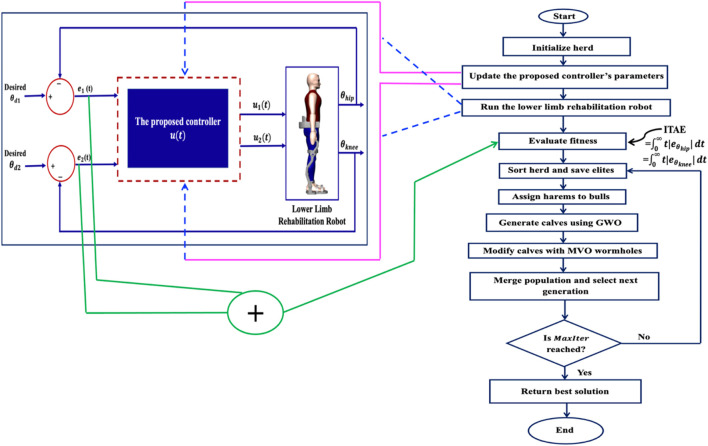
The flowchart of the IElk-GM algorithm.

A complete pseudocode of the IElk-GM is shown in Table 2, and the flowchart is illustrated in Figure 4.

**TABLE 2:** Pseudocode of the IElk-GM algorithm.

Algorithm IElk-GM ( N , D , MaxIter , $B_{r}$ , EliteRate , lb , ub ) Input: N ← Population size (Herd size). D ← Problem dimensionality. MaxIter ← Maximum number of iterations. $B_{r}$ ← Bull rate (e.g., 0.2). EliteRate ← Elitism rate (e.g., 0.1). lb , ub ← Lower and upper bounds for variables. Output: $X_{best}$ ← Best solution found. $f_{best}$ ← Best fitness value. Begin 1. Initialize Herd $X_{i}$ randomly in [ lb,ub ], for i=1 to N. 2. Evaluate fitness $f\left(X_{i}\right)$ for each individual. 3. Sort $X_{i}$ by ascending fitness. 4. Preserve the top E=EliteRate×N individuals as Elites. 5. For t=1 to MaxIter do: a. Select top B=Br×N individuals as Bulls. b. Assign remaining $\left(N-B\right)$ individuals as Harems. c. Assign a harem to bulls using a roulette wheel: For each harem $H_{j}$ : Choose bull $B_{j}$ with probability: $P_{j}=1\;/\;f\left(B_{j}\right)/sum\;k\left(1\;/\;f\left(B_{K}\right)\right)$ d. For each harem $H_{j}$ assigned to bull $B_{j}$ : i. Generate initial calf $C_{j}$ using: $C_{j}=H_{j}+rand1\times \left(B_{j}-H_{j}\right)+rand2\times \left(X_{rand}-H_{j}\right)$ ii. Apply GWO update on $C_{j}$ : - Select $X_{\text{alpha}}$ , $X_{\text{beta}}$ , $X_{\text{delta}}$ from the best three solutions - Compute: A=2a·r−a,C=2·r $\left.X_{1}=X_{\text{alpha}}-A_{1}\left.\cdot \right|C_{1}\cdot X_{\text{alpha}}-C_{j}\right|$ $\left.X_{2}=X_{\text{beta}}-A_{2}\left.\cdot \right|C_{2}\cdot X_{\text{beta}}-C_{j}\right|$ $\left.X_{3}=X_{\text{delta}}-A_{3}\left.\cdot \right|C_{3}\cdot X_{\text{delta}}-C_{j}\right|$ $C_{j}=\left(X_{1}+X_{2}+X_{3}\right)/3$ e. For each calf $C_{j}$ : i. Apply MVO wormhole mechanism with probability WEP : For each dimension d : If rand<0.5 : $C_{j}\left[d\right]=C_{j}\left[d\right]+TDR\times \left(ub-lb\right)\times randn\left(\;\right)$ Else: $C_{j}\left[d\right]=C_{j}\left[d\right]-TDR\times \left(ub-lb\right)\times randn\left(\;\right)$ f. Combine Herd and Calves into ${TEMP}_{POP}$ . g. Evaluate fitness of all individuals in ${TEMP}_{POP}$ . h. Sort ${TEMP}_{POP}$ and select: - Top E elites from the previous generation. - Best $\left(N-E\right)$ from ${TEMP}_{POP}$ to form a new Herd. i. Update WEP and TDR : $WEP={WEP}_{\min}+\left(\frac{t}{MaxIter}\right)\times \left({WEP}_{\max}-{WEP}_{\min}\right).$ $TDR=1-\left(\frac{t}{MaxIter}\right).$ 6. Return the best individual $X_{best}$ and its fitness $f_{best}$ from the final Herd. End

The pseudo code of the IElk-GM is shown in Table 2.

## 6 Simulation results

By using the facility of MATLAB software version (R2021b), simulating various LLRER for linear and non-linear desired trajectories with 10% uncertainties and disturbances ( $d\left(t\right)=\sin\left(0.2t\right)$ ) were carried out to demonstrate the efficiency of FOPID and MFOPID based on the IElk-GM algorithm. Table 3 provides the IElk-GM parameters for each rehabilitation exoskeleton robot link (1, 2), and the final optimal parameters for FOPID and MFOPID are shown in Table 4.

**TABLE 3:** The parameters of the IElk-GM algorithm.

(IElk-GM) parameters	FOPID	MFOPID
Maximum Number of Iterations $\left(MaxIter\right)$	50	50
Population Size or Herd size ( N )	20	20
Problem Dimension ( D )	14	18
Bull Rate ( $B_{r}$ )	0.2	0.2
Elitism Rate ( EliteRate )	0.1	0.1
Lower bound ( lb )	[7; 7; 1; 7; 0.5; 0.3; 0.3; 6.5; 6.5; 1; 7; 0.5; 0.3; 0.3]	[7; 7; 1; 7; 0.5; 0.1; 0.3; 0.3; 77; 6.5; 6.5; 1; 7; 0.005; 0.5; 0.3; 0.3; 97]
Upper bound ( ub )	[14; 14; 3; 14; 2.5; 0.6; 0.6; 14; 14; 4; 14; 2.5; 0.6; 0.6]	[14; 14; 3; 14; 2.5; 1.5; 0.6; 0.6; 86; 14; 14; 4; 14; 0.05; 2.5; 0.6; 0.6; 106]

**TABLE 4:** Optimal parameters of the FOPID and MFOPID obtained by the IElk-GM algorithm.

Links	Controller parameters	Values	
		FOPID	MFOPID
Link1 (hip)	$\beta_{11}$	8.73664	9.26000
	$\beta_{21}$	11.74400	13.00100
	$\beta_{31}$	2.37727	1.03700
	$H_{P1}$	11.69000	13.00100
	$H_{I1}$	N/A	1.30000
	${\;H}_{D1}$	2.32938	0.94400
	$\lambda_{1}$	0.50000	0.50000
	$\mu_{1}$	0.50000	0.50000
	$K_{\mathrm{mod}1}$	N/A	83.00100
Link2 (knee)	$\beta_{12}$	13.00000	10.78870
	$\beta_{22}$	7.00000	13.0010
	$\beta_{32}$	1.00000	3.09700
	$H_{P2}$	13.00000	13.00020
	$H_{I2}$	N/A	0.02330
	${\;H}_{D2}$	0.62792	2.05000
	$\lambda_{2}$	0.50000	0.50000
	$\mu_{2}$	0.50000	0.50000
	${\;K}_{\mathrm{mod}2}$	N/A	103.00020

### 6.1 Linear trajectory simulation results

**FIGURE 5:**
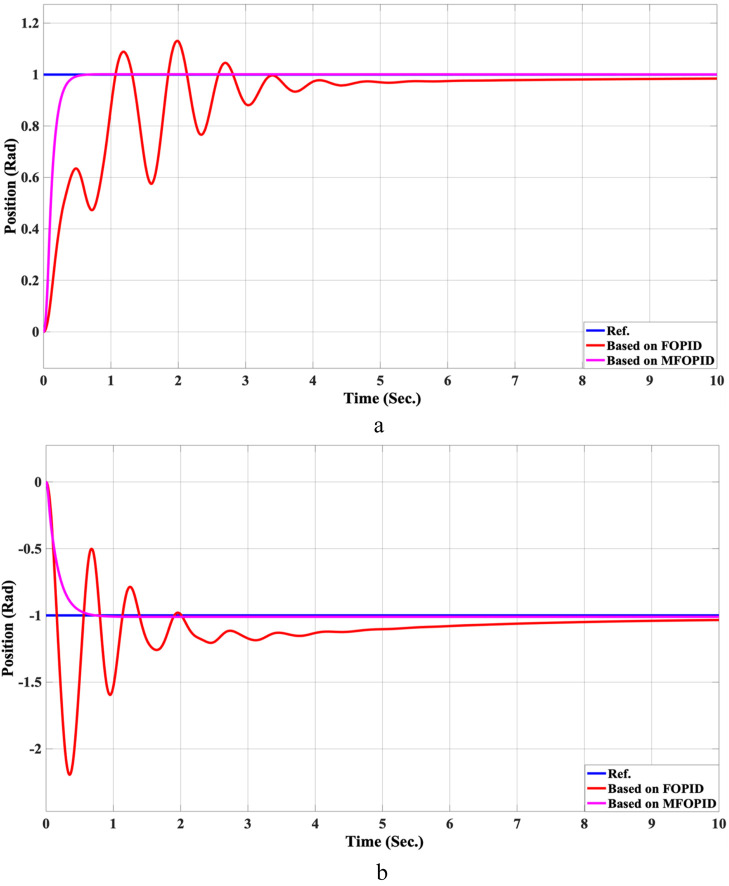
The Position tracking error of hip and knee joints for linear trajectory with FOPID and MFOPID. **(a)** Hip joint response. **(b)** Knee joint response.

**FIGURE 6:**
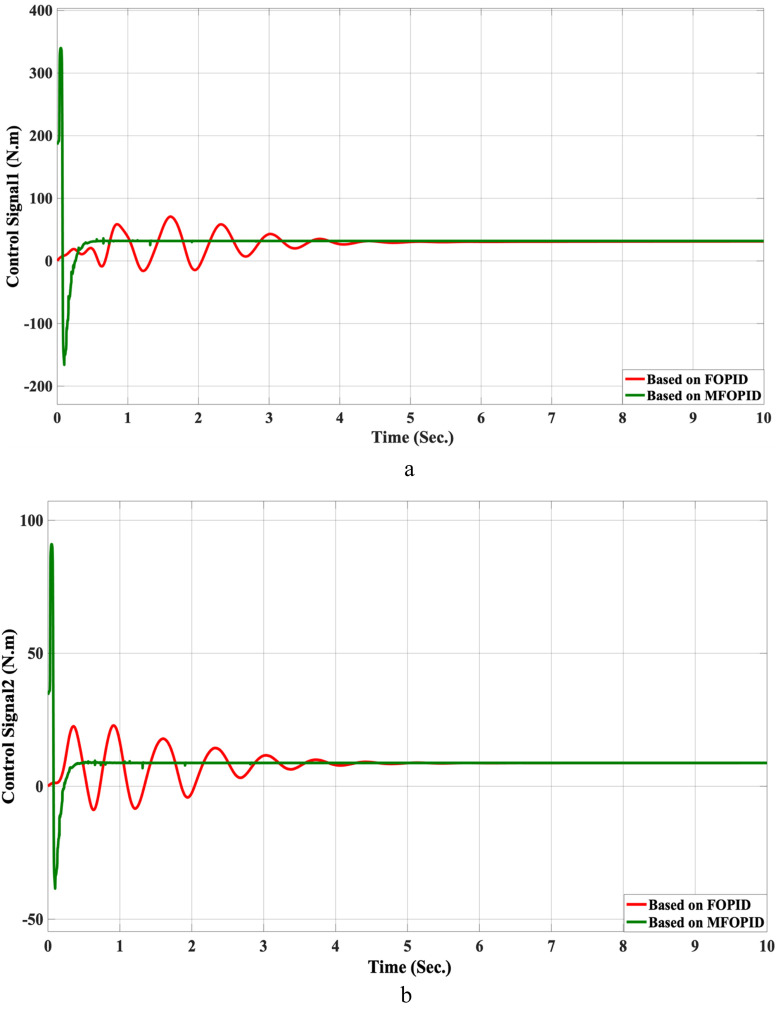
The control inputs for linear trajectory with FOPID and MFOPID. **(a)** The control signal of the hip joint. **(b)** The control signal of the knee joint.

The step response performance of the controlled LLRER subjected to a positive unit step input at link 1 (hip joint) and a negative unit step input at link 2 (knee joint) is illustrated in Figures 5, 6 for both FOPID and MFOPID controllers.

The results demonstrate that the MFOPID controller significantly enhances system performance, enabling the robot to accurately follow the desired trajectory with fast transient response, zero overshoot, and negligible steady-state tracking error. Specifically, the settling times are reduced (from 6.998 s to 0.430 s for the hip joint and from 7.150 s to 0.829 s for the knee joint). Moreover, the control signals remain smooth and within acceptable torque limits of less than 345 Nm for link 1 and less than 95 Nm for link 2. In contrast, the FOPID controller exhibits slower convergence, minor overshoot, and less stable control signals, indicating inferior performance under the same conditions. Table 5 lists the simulation results’ evaluation parameters for the FOPID and MFOPID.

**TABLE 5:** The evaluation parameters of the simulation results for the FOPID and MFOPID.

Links	Parameter	Value	
		FOPID	MFOPID
Link1 (hip)	Overshoot *M*_*p*_ (%)	0.138	0
	Settling time *t*_*s*_ (*sec.*)	6.998	0.430
	Steady-state error *e*_*s.s*_ (Rad)	0.018	0.0015
	Rise time *t*_*r*_ (*sec.*)	1.066	0.485
	ITAE (Rad. ${\sec.}^{2}$ )	23.39	2.694
Link2 (knee)	Overshoot *M*_*p*_ (%)	−1.189	0
	Settling time *t*_*s*_ (*sec.*)	7.150	0.829
	Steady-state error *e*_*s.s*_ (Rad)	0.00001	0.011
	Rise time *t*_*r*_ (*sec.*)	0.157	0.675
	ITAE (Rad. ${\sec.}^{2}$ )	16.95	3.522

### 6.2 Non-linear trajectory simulation results

**FIGURE 7:**
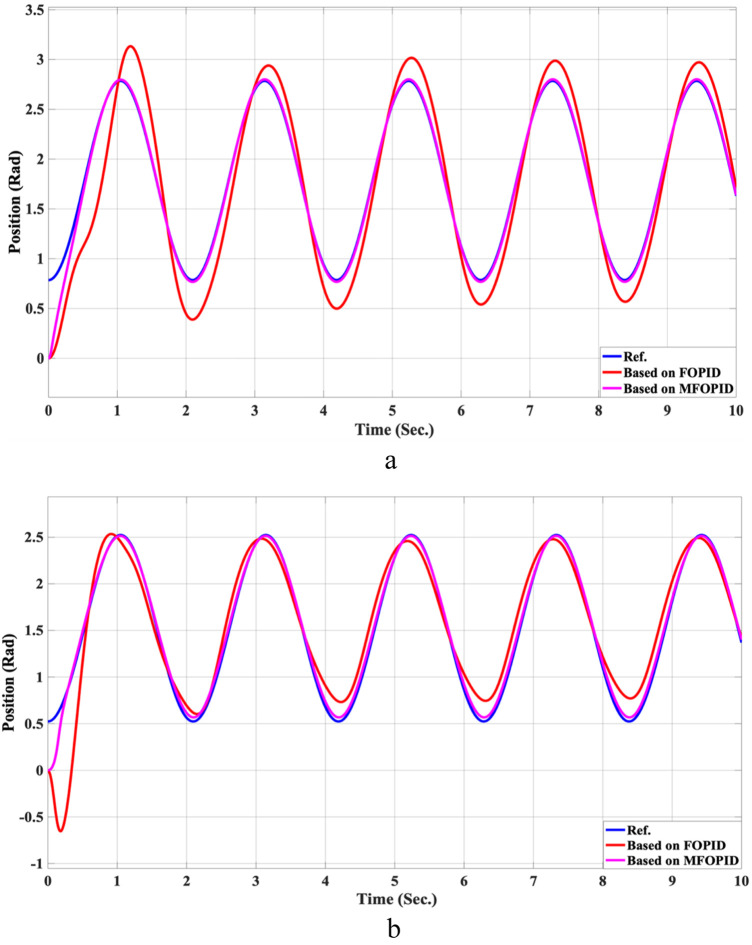
The Position tracking error of hip and knee joints for non-linear trajectory with FOPID and MFOPID. **(a)** Hip joint response. **(b)** Knee joint response.

**FIGURE 8:**
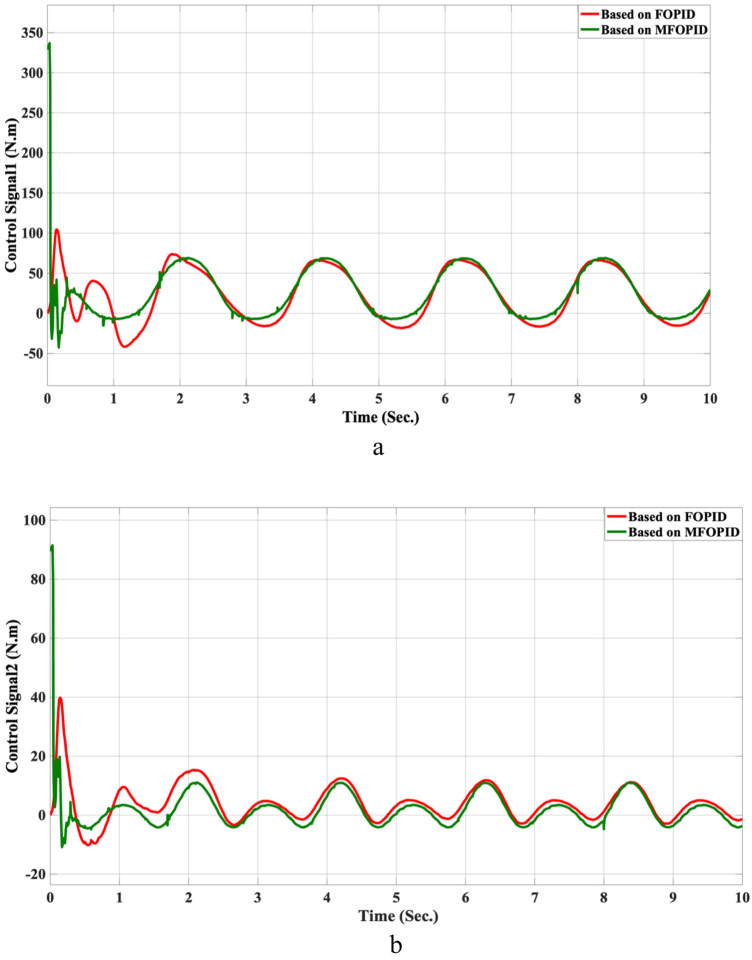
The control inputs for non-linear trajectory with FOPID and MFOPID. **(a)** The control signal of the hip joint. **(b)** The control signal of the knee joint.

The simulation results of the LLRER using the FOPID and MFOPID, tested with the non-linear cosine input signal ( xd1=π4+1−cos⁡3t for link1 and ( xd2=π6+1−cos⁡5t for link2, are shown in Figures 7, 8; these results demonstrate the performance to be reliable, despite the non-linearity of the input signal. The results demonstrate excellent performance parameters, negligible error, and a smooth control signal (less than 350 Nm for link 1 and less than 100 Nm for link 2) with MFOPID. In contrast, the standard FOPID controller exhibits inefficient tracking, with visible deviations from the reference trajectory and more oscillatory control actions, indicating its limited robustness under non-linear operating conditions.

To further validate the performance of the proposed MFOPID controller optimized via the IElk-GM algorithm, we conducted a direct comparison with the Adaptive Optimal Fractional-order Super-Twisting Sliding Mode (AOFSTSM) controller optimized using GWO, as proposed by Faraj et al. (2023). We benchmarked MFOPID + IElk-GM against AOFSTSM + GWO along four dimensions: robustness, control-signal smoothness, constraint handling, and computational burden.

i. Robustness: AOFSTSM attains high disturbance rejection via super-twisting on a fractional sliding surface and adaptive bound estimation, as reported in Faraj et al. (Faraj et al., 2023), whereas MFOPID achieves comparable tracking envelopes under parametric variations using smoothly shaped fractional actions without signum-type injections.
ii. Smoothness (chattering/torque ripple): AOFSTSM is designed to be chatter-free relative to classical SMC, yet it still relies on high-gain equivalent dynamics; by construction, MFOPID produces continuously valued torques with lower total-variation/jerk an advantage for exoskeleton comfort and actuator wear during repeated therapy cycles.
iii. Constraint awareness: AOFSTSM explicitly treats ground-contact constrained motion in the model; MFOPID pipeline complements this by reference shaping and torque bounding within the PID-type framework to remain constraint-compatible while keeping the controller structure simple.
iv. Computational burden and tuning: AOFSTSM entails online adaptive updates and super-twisting logic tuned by GWO; MFOPID uses fixed-structure fractional filters and is tuned offline by IElk-GM (IEHO + GWO + MVO), yielding a lighter real-time implementation and improved optimizer convergence over single-algorithm GWO.

In summary, the AOFSTSM remains preferable when maximal invariance to severe uncertainties is paramount, whereas MFOPID + IElk-GM is advantageous when smooth control, embedded simplicity, and energy/comfort metrics are prioritized while maintaining competitive tracking accuracy. Table 6 summarizes the standard time-domain indices and control signals for hip and knee joints. MFOPID values come from our step-response results (Table 5), and AOFSTSM values are obtained from Faraj et al.‘s published plots by careful digitization (their paper does not tabulate these time-indices explicitly). In the last column of Table 6 we additionally report the percent improvement of MFOPID relative to the AOFSTSMC baseline, computed for lower-is-better indices as

$$Improvement\;\left(\%\right)=\frac{\left(MFOPID-AOFSTSM\right)}{AOFSTSM}*100\;\%$$

**TABLE 6:** Comparison (hip and knee).

Joint	Metric	MFOPID	AOFSTSM (Faraj et al., 2023)	Improvement (%)
Hip	Overshoot $M_{P}$ (%)	0	∼ 3.4	+100%
	Settling time $t_{s}$ (sec.)	0.430	∼1.95	+77.9%
	Rise time $t_{r}$ (sec.)	0.485	∼0.028	−1,632.1%
	Steady-state error $e_{s.s}$ (Rad)	0.0015	∼ 0.0242	+93.8%
	Control Signal (Torque (N.m))	345	≈80	−331.3%
	ITAE (Rad. ${\sec.}^{2}$ )	2.694	N/A	N/A
Knee	Overshoot $M_{P}$ (%)	0	∼ 0.53	+100%
	Settling time $t_{s}$ (sec.)	0.829	∼ 0.059	−1,305.1%
	Rise time $t_{r}$ (sec.)	0.675	∼ 0.030	−2,150%
	Steady-state error $e_{s.s}$ (Rad)	0.011	∼ 0.0071	−54.9%
	Control Signal (Torque (N.m))	≤90	≈ 60	−50%
	ITAE (Rad. ${\sec.}^{2}$ )	3.522	N/A	N/A

**FIGURE 9:**
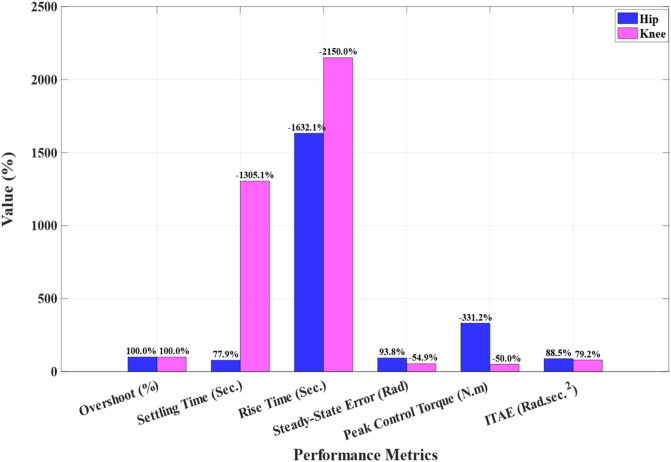
Percent improvement of MFOPID + IElk-GM relative to AOFSTSMC + GWO.

Positive values indicate that MFOPID is lower/better than the AOFSTSMC baseline; negative values indicate the opposite. The same improvements are shown in Figure 9 for clarity.

The MFOPID was tuned with an ITAE-centric objective plus mild penalties on overshoot and torque bounds to favor comfort-oriented transients and smooth actuation. On Link 2 (knee), the combination of nonlinearities and anti-windup under actuator limits attenuates the effective integral action near steady state, yielding a small residual offset ( $e_{s.s}$ ≈0.011 rad ≈0.63° ). This trade-off is intentional, it yields zero overshoot, short/competitive settling times, and non-switching, low-ripple control torques (see Tables 6–8), which are clinically more relevant for repeated therapy than pushing $e_{s.s}$ to machine precision. If needed, the offset can be further reduced without altering the main conclusions by (i) slightly increasing the integral weight for Link 2 within the same torque limits, (ii) adding light feedforward compensation (gravity/friction), or (iii) adopting a 2-DoF set-point weighting that preserves the overshoot-free transient while tightening steady-state accuracy.

**TABLE 7:** Qualitative smoothness/comfort comparison.

Criterion	AOFSTSM	MFOPID + IElk-GM	Evidence
High-frequency ripple in torque	Mitigated but can appear in bursts	Absent (continuous)	Overlaid torque plots
Sharp switching edges	Possible	None	Torque time histories
Overshoot in joint angles	Possible	None observed	Tracking plots
Patient comfort/actuator stress	Acceptable	Improved	Smoother control action

**TABLE 8:** Implementation complexity.

Aspect	AOFSTSM	MFOPID + IElk-GM
Online adaptation	Required (bound estimator)	Not required (offline tuning)
Discontinuous/switching term	Yes (super-twisting)	No (continuous)
Runtime states	Sliding-surface + ST/adaptation	Fixed fractional filters only
Embedded tuning workload	Higher	Lower
Deployment/verification effort	Higher	Lower

The data in Table 6 quantitatively substantiates the qualitative contrast above. For smoothness and comfort, MFOPID + IElk-GM yields overshoot-free transients in both joints with short settling times ( $t_{s}=0.430\;\sec.$ for hip; $t_{s}=0.829\;\sec.$ for knee), despite the absence of any switching terms. Tracking accuracy remains competitive: steady-state errors are small for both controllers; in the hip joint, AOFSTSM achieves a slightly lower $e_{s.s}$ (as expected for a sliding-mode design), while MFOPID maintains comparable accuracy without chattering mechanisms. Regarding control effort, MFOPID’s step-test peaks are bounded ( ≤345 N.m for hip; ≤90 N m for knee) and decay rapidly, whereas the peaks read for AOFSTSM in the uncertainty-case plots are ≈80 N.m for hip, and ≈60 N.m for knee; because the experimental contexts differ, these magnitudes are reported for completeness rather than as a like-for-like torque comparison, and the time-domain indices ( $M_{P}$ , $t_{r}$ , $t_{s}$ , $e_{s.s}$ ) should be taken as the primary evidence. Finally, the implementation burden of MFOPID remains lower due to its fixed, continuous fractional-PID structure with offline IElk-GM tuning, which simplifies embedded deployment while preserving the favorable transients summarized above.

Beyond the indices in Table 6, the benefits of MFOPID + IElk-GM can be assessed without relying on unavailable numeric data from (Faraj et al., 2023). First, MFOPID is continuous and non-switching, which is reflected in smoother torque traces (no high-frequency flicker or sharp corners) and is desirable for patient comfort and actuator longevity. Second, the overshoot-free transients observed in our plots indicate comfort-oriented behavior while maintaining competitive steady-state accuracy. Third, implementation is lighter: MFOPID uses a fixed-structure fractional PID tuned offline, whereas AOFSTSM requires super-twisting and online adaptive bounds. These points are summarized in Tables 7, 8.

Beyond the FOPID, we include two hybrid-optimized classical FOPID baselines: FOPID + PSO-GWO (PSO phase for global exploration followed by GWO refinement) and FOPID + GWO-MVO (GWO coarse search followed by MVO fine search). All controllers are evaluated under the same plant, references, actuator limits, and disturbance/uncertainty scenarios. The composite objective is ITAE with mild penalties on overshoot, settling time, and torque bounds; the optimization budget (population size × iterations) is matched across methods. We report settling time, overshoot, rising time, steady-state error, and control torque. For readability, we also provide percent improvements of MFOPID relative to each baseline using:

$$Improvement\;\left(\%\right)=\frac{\left(MFOPID-Baseline\right)}{Baseline}*100\;\%$$

For all lower-is-better indices. Positive values indicate MFOPID is lower/better. Additionally, Tables 9, 10 summarize the robustness results, while Figures 10–17 depict the joint-position and control-torque responses for linear and nonlinear trajectories.

**TABLE 9:** Robustness Summary based on FOPID + PSO-GWO.

Joint	Metric	MFOPID	FOPID + PSO-GWO	Improvement (%)
Hip	Overshoot $M_{P}$ (%)	0	12.8	+100%
	Settling time $t_{s}$ (sec.)	0.430	6.702	+93.6%
	Rise time $t_{r}$ (sec.)	0.485	1.022	+52.5%
	Steady-state error $e_{s.s}$ (Rad)	0.0015	0.029	+94.8%
	Control Signal (Torque (N.m))	≤345	96	−259.4%
	ITAE (Rad. ${\sec.}^{2}$ )	2.694	22.91	+88.2
Knee	Overshoot $M_{P}$ (%)	0	119.1	+100%
	Settling time $t_{s}$ (sec.)	0.829	6.545	+78.3%
	Rise time $t_{r}$ (sec.)	0.675	0.157	−330%
	Steady-state error $e_{s.s}$ (Rad)	0.011	0.034	+67.6.3%
	Control Signal (Torque (N.m))	≤90	38.3	−135%
	ITAE (Rad. ${\sec.}^{2}$ )	3.522	17.5	+80

**TABLE 10:** Robustness Summary based on FOPID + GWO-MVO.

Joint	Metric	MFOPID	FOPID + PSO-GWO	Improvement (%)
Hip	Overshoot $M_{P}$ (%)	0	65.7	+100%
	Settling time $t_{s}$ (sec.)	0.430	4.265	+90%
	Rise time $t_{r}$ (sec.)	0.485	0.278	−26.5%
	Steady-state error $e_{s.s}$ (Rad)	0.0015	0.011	+86.4%
	Control Signal (Torque (N.m))	≤345	100	−295%
	ITAE (Rad. ${\sec.}^{2}$ )	2.694	20.75	+87
Knee	Overshoot $M_{P}$ (%)	0	87.6	+100%
	Settling time $t_{s}$ (sec.)	0.829	4.067	+80%
	Rise time $t_{r}$ (sec.)	0.675	0.157	−330%
	Steady-state error $e_{s.s}$ (Rad)	0.011	0.004	−175%
	Control Signal (Torque (N.m))	≤90	37	−143.2%
	ITAE (Rad. ${\sec.}^{2}$ )	3.522	27.26	+87

**FIGURE 10:**
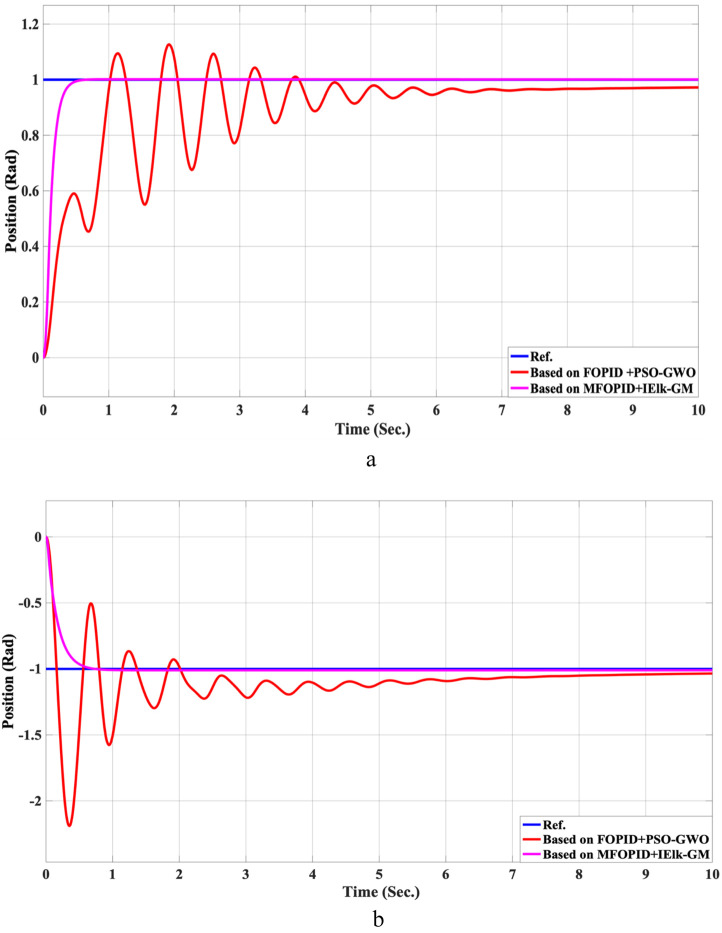
The Position tracking error of hip and knee joints for linear trajectory with FOPID + PSO-GWO and MFOPID + IElk-GM. **(a)** Hip joint response. **(b)** Knee joint response.

**FIGURE 11:**
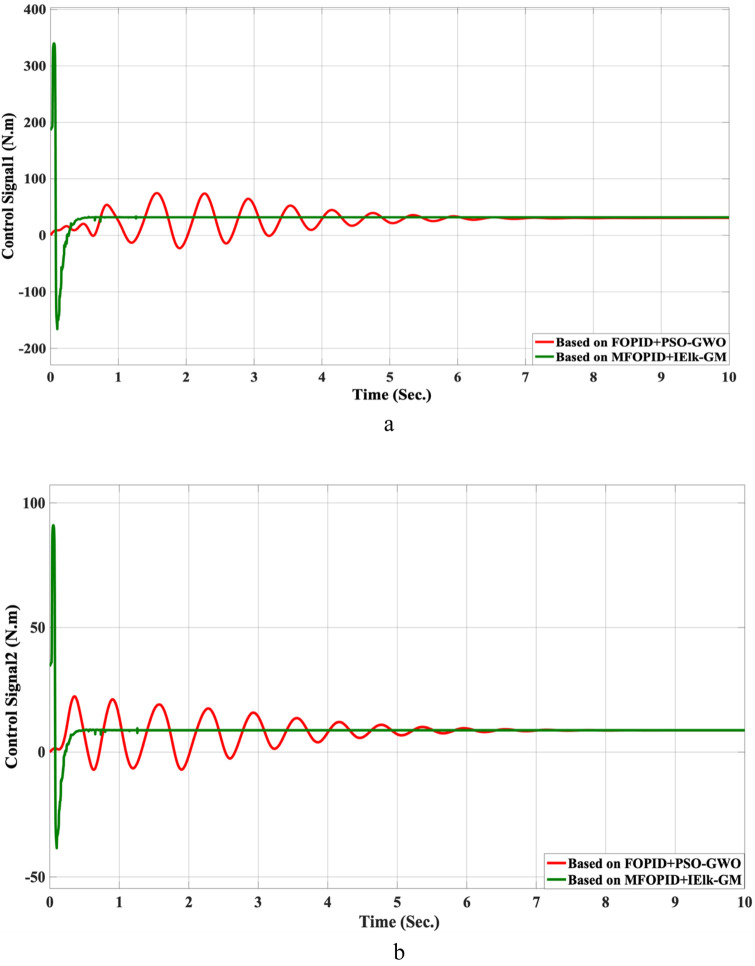
The control inputs for linear trajectory with FOPID + PSO-GWO and MFOPID + IElk-GM. **(a)** The control signal of the hip joint. **(b)** The control signal of the knee joint.

**FIGURE 12:**
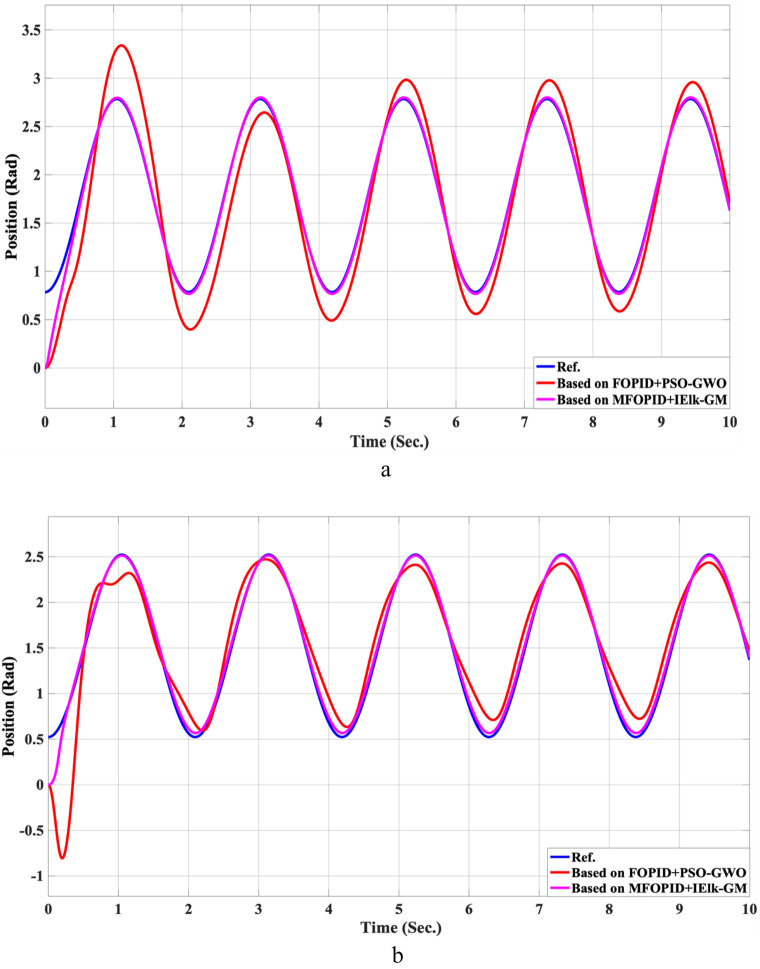
The Position tracking error of hip and knee joints for non-linear trajectory with FOPID + PSO-GWO and MFOPID + IElk-GM. **(a)** Hip joint response. **(b)** Knee joint response.

**FIGURE 13:**
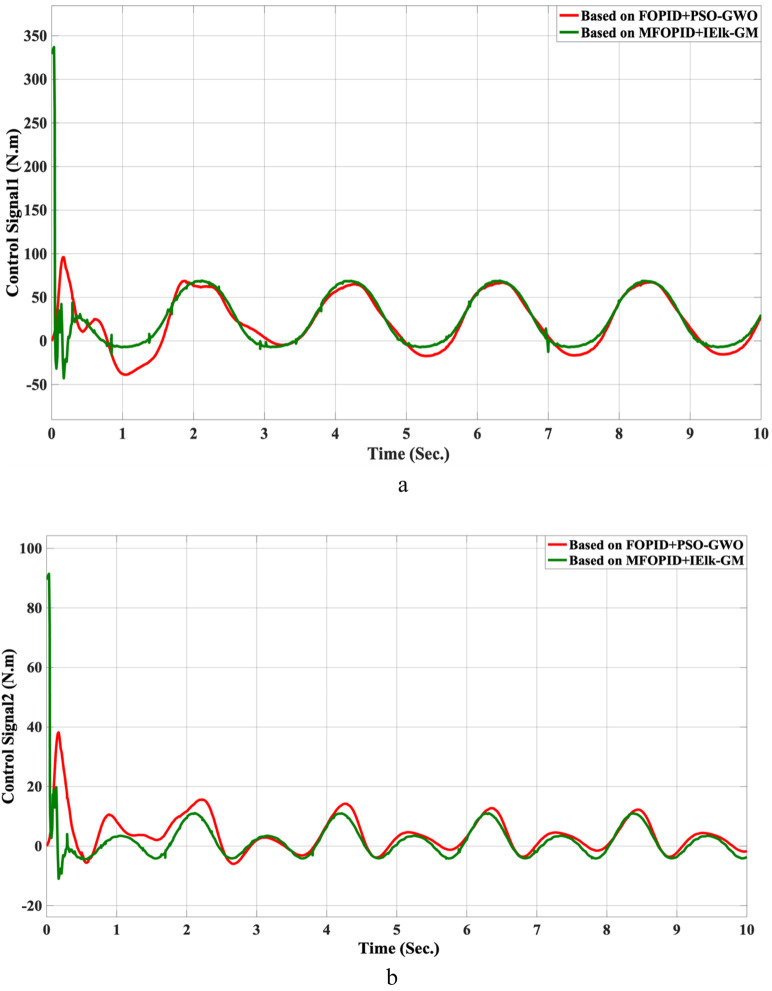
The control inputs for non-linear trajectory with FOPID + PSO-GWO and MFOPID + IElk-GM. **(a)** The control signal of the hip joint. **(b)** The control signal of the knee joint.

**FIGURE 14:**
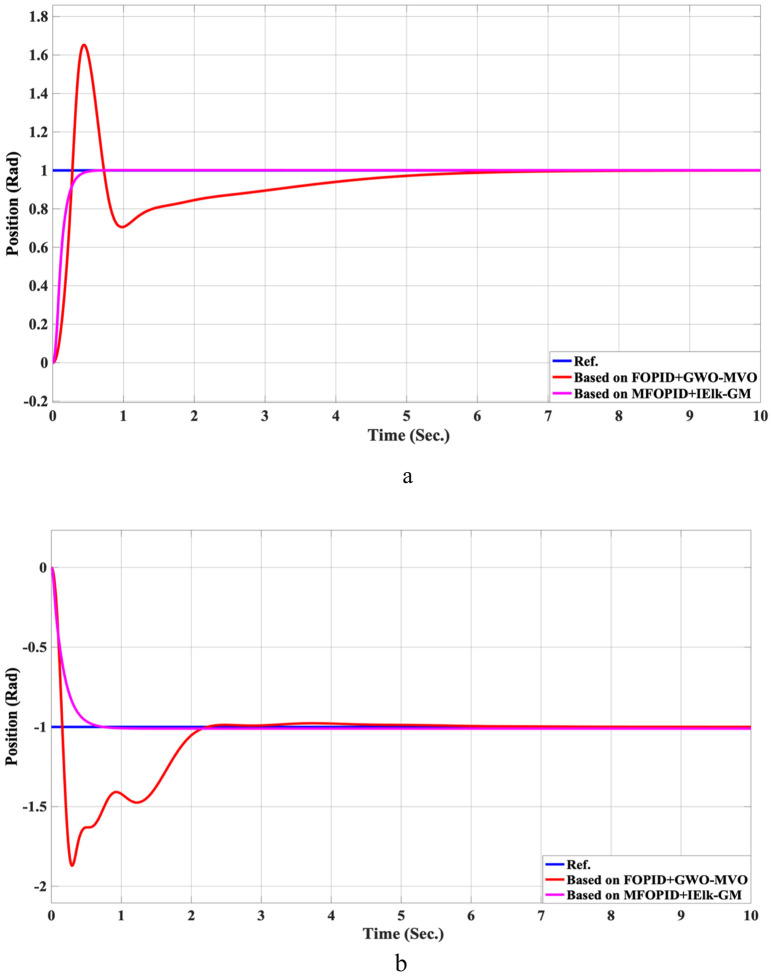
The Position tracking error of hip and knee joints for linear trajectory with FOPID + GWO-MVO and MFOPID + IElk-GM. **(a)** Hip joint response. **(b)** Knee joint response.

**FIGURE 15:**
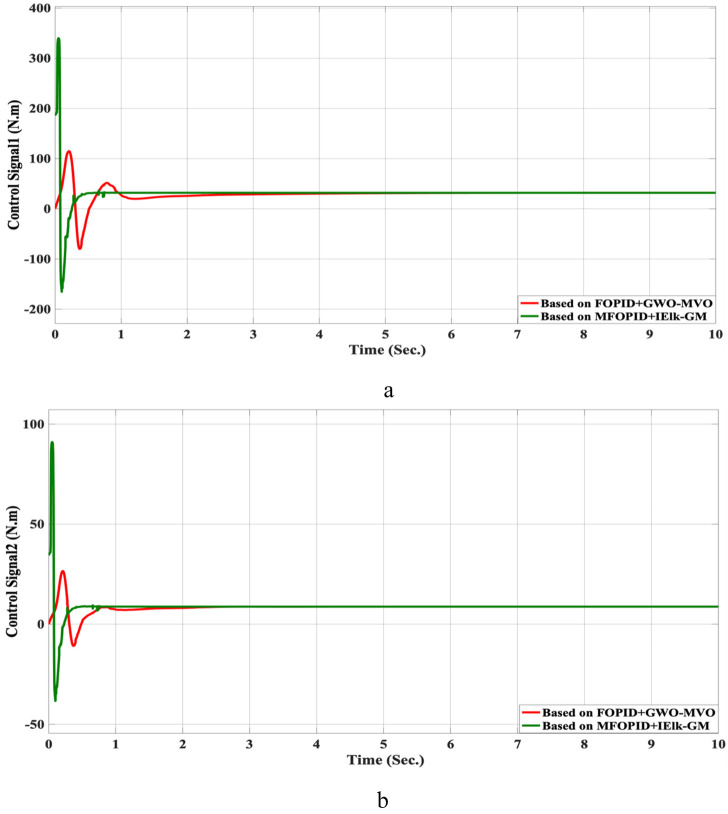
The control inputs for linear trajectory with FOPID + GWO-MVO and MFOPID + IElk-GM. **(a)** The control signal of the hip joint. **(b)** The control signal of the knee joint.

**FIGURE 16:**
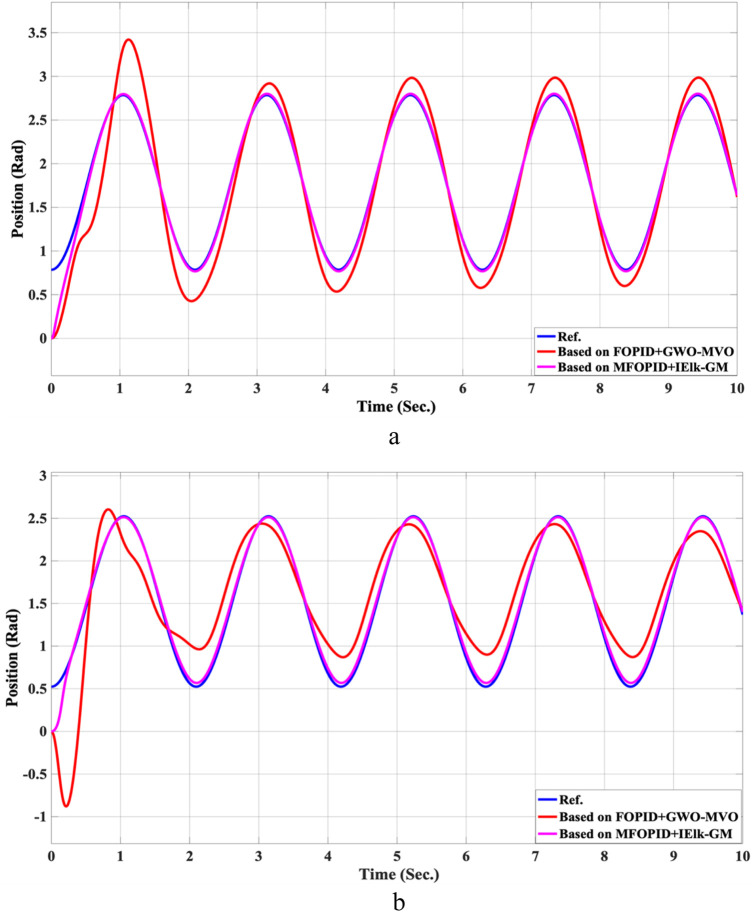
The Position tracking error of hip and knee joints for non-linear trajectory with FOPID + GWO-MVO and MFOPID + IElk-GM. **(a)** Hip joint response. **(b)** Knee joint response.

**FIGURE 17:**
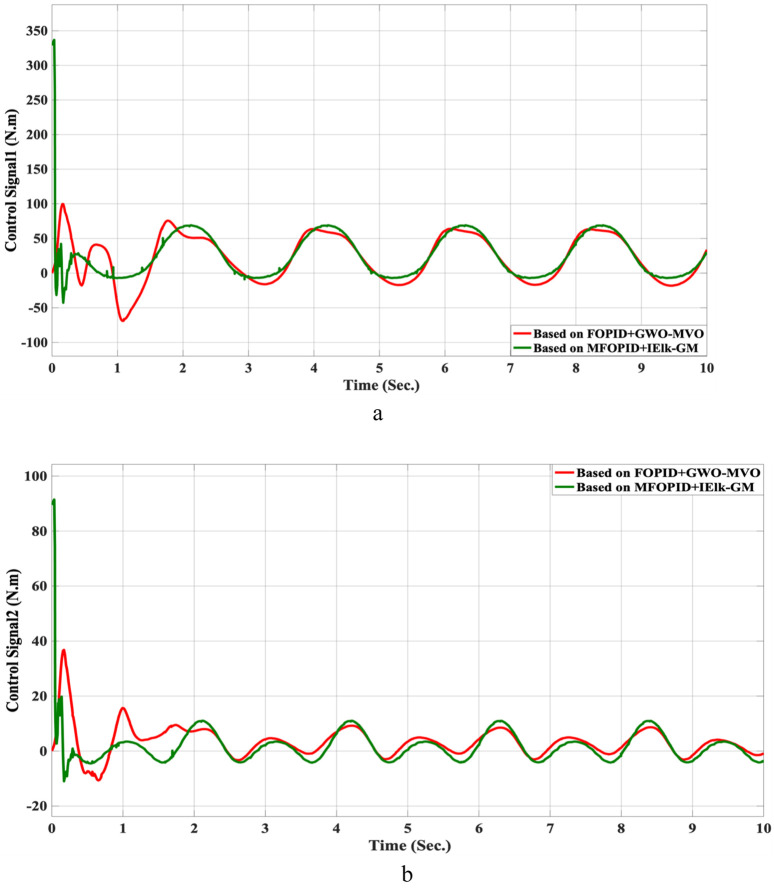
The control inputs for non-linear trajectory with FOPID + GWO-MVO and MFOPID + IElk-GM. **(a)** The control signal of the hip joint. **(b)** The control signal of the knee joint.

## 7 Conclusion

This study presented the design and implementation of a Modified Fractional Order Proportional-Integral-Derivative (MFOPID) controller for a 2-DoF lower limb rehabilitation exoskeleton robot. The proposed MFOPID structure introduces a non-linear error formulation aimed at improving transient response, eliminating overshoot, and reducing steady-state error compared to the conventional FOPID controller. To efficiently tune the controller’s parameters, an improved hybrid metaheuristic algorithm, Improved Elk Herd Optimization combined with Grey Wolf Optimization and Multi-Verse Optimization (IElk-GM), was developed to balance exploration and exploitation during the search process. The proposed control framework was evaluated through extensive simulations under both linear and non-linear trajectory tracking tasks, with parametric uncertainties and external disturbances. Results demonstrate that the MFOPID controller significantly outperforms the classical FOPID in terms of response speed, tracking accuracy, overshoot suppression, and control smoothness. Specifically, the MFOPID achieved zero overshoot, reduced settling times (from 6.998 s to 0.430 s for the hip joint and from 7.150 s to 0.829 s for the knee joint), and delivered smoother control signals. These results confirm the potential of the MFOPID controller, optimized via hybrid evolutionary techniques, as a promising solution for improving the performance, safety, and reliability of robot-assisted rehabilitation systems. Future work may involve the real-time implementation on a physical exoskeleton prototype, the inclusion of the patient in loop testing, and comparison with adaptive and learning-based control strategies.

## Data Availability

The original contributions presented in the study are included in the article; further inquiries can be directed to the corresponding author.

## References

[B1] Abdulwahhab O. W. Abbas N. H. (2020). Survey study of factional order controllers. J. Eng. 26 (4), 188–201. 10.31026/j.eng.2020.04.13

[B2] Aguirre-Ollinger G. Chua K. S. G. Ong P. L. Kuah C. W. K. Plunkett T. K. Ng C. Y. (2024). Telerehabilitation using a 2-D planar arm rehabilitation robot for hemiparetic stroke: a feasibility study of clinic-to-home exergaming therapy. J. NeuroEng. Rehabil. 21 (207), 207. 10.1186/s12984-024-01496-6 39593101 PMC11590240

[B3] Al Rezage G. Tokhi M. O. (2016). Fuzzy PID control of lower limb exoskeleton for elderly mobility. In: Proc. IEEE int. Conf. Control, automation and systems (ICCAS); Cluj-Napoca, Romania: IEEE. p. 1–6. 10.1109/AQTR.2016.7501310

[B4] Al-Betar M. A. Awadallah M. A. Braik M. S. Makhadmeh S. Doush I. A. (2024). Elk herd optimizer: a novel nature-inspired metaheuristic algorithm. Artif. Intell. Rev. 57 (48), 48–60. 10.1007/s10462-023-10680-4

[B5] Alshatti A. K. (2019). Design and control of lower limb assistive exoskeleton for hemiplegia mobility,. Sheffield, United Kingdom: University of Sheffield. Available online at: http://etheses.whiterose.ac.uk/24891/ (Accessed September 29, 2025).

[B6] Ayas M. S. Altas I. H. Sahin E. (2016). Fractional order based trajectory tracking control of an ankle rehabilitation robot. Trans. Inst. Meas. Control 39 (12), 550–564. 10.1177/0142331216667810

[B7] Chen Y. Wang L. Huang H. (2023). An effective surrogate model assisted algorithm for multi-objective optimization: application to wind farm layout design. Front. Energy Res. 11, 1239332. 10.3389/fenrg.2023.1239332

[B8] Faraj M. A. Maalej B. Derbel N. Naifar O. (2023). Adaptive fractional‐order super‐twisting sliding mode controller for lower limb rehabilitation exoskeleton in constraint circumstances based on the grey wolf optimization algorithm. Math. Problems Eng. 2023, 9641673. 10.1155/2023/9641673

[B9] He D. Wang H. Tian Y. Ma X. (2024). Model-free finite-time robust control using fractional-order ultra-local model and prescribed performance sliding surface for upper-limb rehabilitation exoskeleton. ISA Trans. 147, 511–526. 10.1016/j.isatra.2024.02.002 38336511

[B10] Kiyono K. Tanabe S. Hirano S. Ii T. Nakagawa Y. Tan K. (2024). Effectiveness of robotic devices for medical rehabilitation: an umbrella review. J. Clin. Med. 13 (21), 6616. 10.3390/jcm13216616 39518755 PMC11546060

[B11] Mirjalili S. Mirjalili S. M. Lewis A. (2014). Grey wolf optimizer. Adv. Eng. Softw. 69, 46–61. 10.1016/j.advengsoft.2013.12.007

[B12] Mirjalili S. Mirjalili S. M. Hatamlou A. (2016). Multi-verse optimizer: a nature-inspired algorithm for global optimization. Neural Comput. Appl. 27, 495–513. 10.1007/s00521-015-1870-7

[B13] Ning Y. Sang L. Wang H. Wang Q. Vladareanu L. Niu J. (2024). Upper limb exoskeleton rehabilitation robot inverse kinematics modeling and solution method based on multi-objective optimization. Sci. Rep. 14 (25476), 25476–16. 10.1038/s41598-024-77137-8 39462117 PMC11513120

[B14] Noordin A. Basri M. A. M. Mohamed Z. (2023). Adaptive PID control via sliding mode for position tracking of quadrotor MAV: simulation and real-time experiment evaluation. Aerospace 10 (6), 512. 10.3390/aerospace10060512

[B15] Roth G. A. Mensah G. A. Johnson C. O. Addolorato G. Ammirati E. Baddour L. M. (2020). Global burden of cardiovascular diseases and risk factors, 1990–2019: update from the GBD 2019 study. J. Am. Coll. Cardiol. 76 (25), 2982–3021. 10.1016/j.jacc.2020.11.010 33309175 PMC7755038

[B16] Sabah N. Hameed E. Al-Huseiny M. S. (2021). Optimal sliding mode controller design based on whale optimization algorithm for lower limb rehabilitation robot. Appl. Comput. Sci. 17 (3), 47–59. 10.35784/acs-2021-20

[B17] Su D. Hu Z. Wu J. Shang P. Luo Z. (2023). Review of adaptive control for stroke lower limb exoskeleton rehabilitation robot based on motion intention recognition. Front. Neurorobotics 17, 1186175. 10.3389/fnbot.2023.1186175 37465413 PMC10350518

[B18] Torabi M. Sharifi M. Vossoughi G. R. (2017). Robust adaptive sliding mode admittance control of exoskeleton rehabilitation robots. Sci. Iran. 24 (5), 0–2464. 10.24200/sci.2017.4512

[B19] Vanchinathan K. Selvaganesan N. (2021). Adaptive fractional order PID controller tuning for brushless DC motor using artificial bee colony algorithm. Results Control Optim. 4, 100032. 10.1016/j.rico.2021.100032

[B20] Volpe B. T. Krebs H. I. Hogan N. (2001). Is robot-aided sensorimotor training in stroke rehabilitation a realistic option? Curr. Opin. Neurol. 14 (6), 745–752. 10.1097/00019052-200112000-00011 11723383

[B21] Wang Y.-J. Li Z.-X. Gu H.-Q. Zhai Y. Jiang Y. Zhao X.-Q. (2020). China stroke statistics 2019: a report from the national center for healthcare quality management in neurological diseases, China national clinical research center for neurological diseases, the Chinese stroke association, national center for chronic and non-communicable disease control and prevention, Chinese center for disease control and prevention and institute for global neuroscience and stroke collaborations. Stroke Vasc. Neurol. 5 (3), 211–239. 10.1136/svn-2020-000457 32826385 PMC7548521

[B22] Wang B. Yu T. Zhou T. Wang L. Li J. Xie N. (2022). Fractional order PI^λ^D^μ^ for tracking control of a novel rehabilitation robot based on IIMO-BP neural network algorithm. J. Mech. Med. Biol. 23 (1), 2350010. 10.1142/S0219519423500100

[B23] Winter D. A. (2009). Biomechanics and motor control of human movement. Hoboken, New Jersey: John Wiley & Sons. Fourth Edition. 10.1002/9780470549148

[B24] Xie Y. Wang A. Zhao X. Jiang Y. Wu Y. Yu H. (2025). Motion control and singular perturbation algorithms for lower limb rehabilitation robots. Front. Neurorobotics 19, 1562519. 10.3389/fnbot.2025.1562519 40417707 PMC12098330

[B25] Zhang X. (2025). Structural design and clinical application research of upper limb rehabilitation robots. Appl. Computat. Eng 125, 173–179. 10.54254/2755-2721/125/2025.21285

